# Efficacy of additional periprostatic apex nerve block on pain in each of 12 transrectal prostate core biopsies: a retrospective study

**DOI:** 10.1186/s12894-021-00898-1

**Published:** 2021-09-16

**Authors:** Jeong Woo Yoo, Kyo Chul Koo, Byung Ha Chung, Kwang Suk Lee

**Affiliations:** Department of Urology, Gangnam Severance Hospital, Yonsei University College of Medicine, 211 Eonju-ro, Gangnam-gu, Seoul, 06273 Republic of Korea

**Keywords:** Biopsy, Nerve block, Pain, Prostate

## Abstract

**Background:**

We identified pain variation according to prostate biopsy sites and compared differences in pain relief according to the site of periprostatic nerve block (PNB).

**Methods:**

This retrospective study collected data from 312 patients who underwent transrectal prostate biopsies between January 2019 and August 2020. Patients were stratified into two groups according to the site of local anesthesia (base vs. base and apex PNB), with each block achieved with 2.5 cm^3^ of 2% lidocaine. Pain scores were assessed using the visual analog scale at the following time points: probe insertion, PNB at base, PNB at apex, each of the 12 core biopsy sites, and 15 min after biopsy. The results were analyzed using a linear mixed model.

**Results:**

The average pain scores were significantly higher in the base-only PNB group than were those in the base and apex PNB group (3.88 vs 2.82, *p* < 0.001). In the base-only PNB group, the pain scores increased from base to apex (*p *< 0.001), and the pain at each site also gradually increased as the biopsy proceeded (*p* < 0.001). In contrast, in the base and apex PNB group, there was minor change in pain scores throughout the procedure.

**Conclusions:**

The pain scores varied at each site during the prostate biopsy. The provision of a base and apex PNB provided greater pain relief than does base-only PNB during prostate biopsy.

## Background

Systematic random prostate biopsy is generally performed via a transrectal or transperineal approach for the diagnosis of prostate cancer [1, 2]. The transrectal ultrasound (TRUS)-guided random prostate biopsy may cause severe pain, hematuria, urinary retention, infection, and even septic shock [3–5]. As the experience of pain during prostate biopsy lowers patient compliance, this procedure should be performed under local anesthesia, in accordance with the guidelines provided by the National Comprehensive Cancer Network (NCCN).

The availability of various anesthetic techniques greatly enhances patients’ acceptability of prostate biopsy. Active pain relief was not previously adopted; however, for pain relief, general anesthesia, intrarectal local anesthesia, pudendal and caudal nerve block, periprostatic local anesthesia, intravenous conscious sedation (propofol, midazolam), and intravenous analgesics (fentanyl) have been attempted [6]. Among these, intrarectal lidocaine gel (IRLG), pelvic plexus block (PPB), and periprostatic nerve block (PNB) using prilocaine or lidocaine are generally used in the clinical setting [7–11].

A previous meta-analysis reported the usefulness of both the PPB and PNB for pain relief during prostate biopsy, while a recent randomized clinical trial conducted by our research group found no difference between the efficacy of the PPB and base PNB [6, 8]. However, in the latter study, the PNB was stratified according to the nerve block site. The administration of a base PNB and an additional apex PNB relieve overall pain as it blocks a sensitive somatic nerve branch of the pudendal nerve [12].

With the consideration that the level of pain differs according to the site at which the PNB is administered, we speculated that pain levels would also differ among random biopsy sites, and that the degree of pain relief would vary at each site following the administration of an additional nerve block. To the best of our knowledge, no prior studies have investigated these issues. Therefore, we aimed to evaluate differences in pain levels among 12 core biopsy sites, and assess pain relief according to the site of PNB administration.

## Methods

### Patient selection

**Fig. 1:**
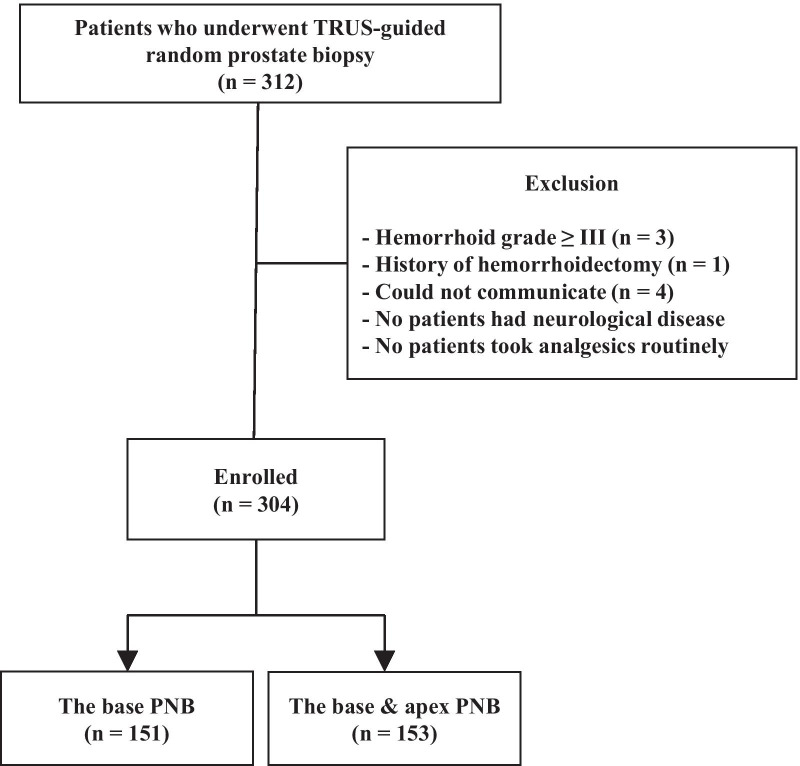
Study cohort flow diagram. PNB, periprostatic nerve block; TRUS, transrectal ultrasound

This retrospective study collected data from 312 consecutive patients who underwent TRUS-guided prostate biopsies at single institution (Gangnam Severance Hospital, Yonsei University Health System) between January 2019 and August 2020. The indication for prostate biopsy was persistent clinical suspicion of prostate cancer due to an elevated prostate-specific antigen (PSA) level (> 2.5 ng/mL) and/or a positive digital rectal examination (DRE) and continuous rise in PSA level during the follow-up period. The exclusion criteria comprised the following: (1) hemorrhoid grade ≥ III (n = 3), which indicated that the hemorrhoid tissue prolapsed beyond the dentate line; (2) history of hemorrhoidectomy (n = 1); and (3) inability to communicate (n = 4; two were foreigners and two were old men with communication difficulties). None of the 312 patients had neurological disease such as paraplegia or hemiplegia and none routinely used analgesics for chronic pain or other reasons. Finally, a total of 304 (97.4%) patients were included in the analysis (Fig. 1). This study was approved by the institutional ethics committee (Yonsei University Health System, Seoul, Korea, 3-2019-0418), and all procedures were conducted in accordance with the ethical standards of the 1964 Helsinki declaration and its later amendments. The requirement for informed consent was waived for this study as it was based on retrospective, anonymous patient data and did not involve patient intervention or the use of human tissue samples.

### Data collection

The collected patient data included age, PSA level, prostate volume, history of prostate biopsy, DRE (positive; hard surface, nodular lesions or mass-like lesion), pathologic results, time of PNB and biopsies, adverse events (vasovagal syncope, allergic reaction, acute urine retention, clot retention, fever), and visual analog scale (VAS; ranging from 0 [no pain] to 10 [worst pain]) score measured at various time points: probe insertion, PNB at base, PNB at apex, each of the 12 core biopsy sites, and 15 min after prostate biopsy.

### TRUS-guided prostate biopsy technique

All patients were hospitalized for half a day. Antibiotics (third generation cephalosporin) were administered prophylactically via intravenous injection 1 h before the biopsy and upon discharge (100 mg orally, three times a day for 2 days).

The patients assumed a left lateral decubitus position during the biopsy. All biopsies were performed by the same experienced urologist. After povidone iodine rectal preparation, all patients received 10 cm^3^ of 2 % IRLG (Instillagel®, Farco-Farma GmbH, Köln, Germany). After 5 min, a transrectal probe was inserted to measure the prostate volume, and the PNB procedure was performed with a Chiba needle (A & A M.D. Inc., Seongnam, Korea).

Biopsies were performed with the BK 3000 ultrasound system (Analogic Corporation, Peabody, MA, USA) using a 7.5–12-MHz multiplanar probe at each of the 12 biopsy sites, in the following order: right lateral base, right lateral mid, right lateral apex, right medial base, right medial mid, right medial apex, left lateral base, left lateral mid, left lateral apex, left medial base, left medial mid, and left medial apex. A 20-cm, 18-gauge disposable core biopsy instrument (Max-Core®, CR Bard Inc., Covington, GA, USA) was used in all cases.

### Site of injection

The two methods of local anesthesia (base PNB and base and apex PNB) were alternately administered—(1) odd days (base-only PNB group: the PNB was administered on both sides of the prostate base) and (2) even days (base and apex PNB group: an additional PNB was administered on both sides of the prostate apex). The site of base injections was aimed at the major neurovascular bundle, after confirming the triangular echogenic “Mount Everest sign” between the base of the prostate and seminal vesicle in the parasagittal longitudinal view of the TRUS [6]. PNB administration at this site was considered to have anesthetized a large portion of the prostate gland. The site for apex injections was aimed at a smaller triangular echogenicity between the puborectalis muscles and the apex of the prostate gland. Each PNB injection utilized 2.5 cm^3^ of 2% lidocaine [6]. The base and apex PNB group received the base injection before the apex injection.

### Study endpoints

The primary study endpoint was the pain scale score for the two PNB methods in each of the 12 core biopsy sites. The secondary endpoint was the comparison of pain scores between the PNB methods.

### Statistical analysis

VAS pain scores in the base and apex PNB were defined as the average of the VAS scores for base and apex injections, respectively. Average pain was defined as the mean (± standard deviation [SD]) of the individual VAS pain scores at the 12 sites. Average pain at the base, mid, and apex of the prostate was defined as the mean (± SD) of the individual VAS pain scores at the base, mid, apex sites, respectively.

Continuous variables are expressed as either the mean ± SD or median (interquartile range). Categorical variables were reported as the number of occurrences and frequency. Comparisons between the base-only PNB group and the base and apex PNB group were performed with the independent t-test for continuous variables, and the chi-square test (Fisher’s exact test) for two or more variables. The results were also analyzed using a linear mixed model and illustrated with a mean profile graph. The correlation matrix structure of the linear mixed model was used to determine the relationship between the measured data at various points in time via the application of compound symmetry. Statistical analyses were performed using SAS (version 9.4.; SAS Institute, Cary, NC, USA) and PASS (version 15; NCSS, LLC. Kaysville, Utah, USA). The level of statistical significance was set at *p* < 0.05.

## Results

### Baseline characteristics and VAS pain scores according to PNB sites

Patient characteristics and VAS pain scores according to PNB sites are presented in Table 1. No differences in PSA concentrations (7.56 ng/mL vs. 6.76 ng/mL), prostate volume (36.55 mL vs. 36.79 mL), or prostate cancer detection rate (62.9 % vs. 62.1 %) were observed between the base-only PNB group and the base and apex PNB group. There were no significant differences in VAS pain scores among the different time points (probe insertion, PNB at base, and 15 min after prostate biopsy). Average pain scores at the base, mid, and apex sites are presented in Table 2. The average VAS pain score across all regions (overall, base, mid, and apex) in the base and apex PNB group was lower than that in the base-only PNB group.

**Table 1:** Baseline characteristics and VAS pain scores according to the PNB sites

	Base	Base and apex	*p*
N	151 (49.7)	153 (50.3)	
Age (years)	67.86 ± 15.74	68.19 ± 8.59	0.740
PSA (ng/mL)	7.56 (5.40–11.59)	6.76 (4.47–10.19)	0.074
Prostate volume (mL)	36.55 ± 15.74	36.79 ± 16.78	0.900
History of prostate biopsy (n, %)	27 (17.9)	19 (12.4)	0.186
Positive DRE (n, %)	35 (35.7)	25 (28.4)	0.287
Prostate cancer detection rate (n, %)	95 (62.9)	95 (62.1)	0.882
*Gleason score*			0.171
6 (n, %)	18 (18.9)	26 (27.4)	
≥ 7 (n, %)	77 (81.1)	69 (72.6)	
*Pain*			
Probe insertion	3.39 ± 2.12	3.36 ± 2.43	0.905
PNB at base	2.91 ± 1.83	2.79 ± 1.94	0.591
PNB at apex		4.60 ± 2.50	
At 15 min post prostate biopsy	0.14 ± 0.58	0.16 ± 0.74	0.749
*Time*			
Periprostatic nerve block (min)	2.42 ± 0.77	2.59 ± 0.88	0.084
Prostate biopsy (min)	4.20 ± 2.59	4.66 ± 5.41	0.343
*Adverse events*			
Vasovagal syncope (n, %)	2 (1.3)	0 (0.0)	0.246
Allergic reaction (n, %)	0 (0.0)	0 (0.0)	–
AUR (n, %)	0 (0.0)	0 (0.0)	–
Urinary retention because of blood clot (n, %)	0 (0.0)	0 (0.0)	–
Fever (n, %)	0 (0.0)	0 (0.0)	–

Data are expressed as number (%), mean ± standard deviation, and median (IQR range)

AUR, acute urinary retention; DRE, digital rectal exam; IQR, interquartile; PNB, periprostatic nerve block; PSA, prostate specific antigen; VAS, visual analog scale

**Table 2:** Mean VAS pain scores in TRUS-guided random prostate biopsy groups according to PNB sites

	Base	Base and apex	*p*
*12 core VAS pain scores*			
Average pain^a^	3.88 ± 2.27	2.82 ± 2.17	< 0.001
Average pain at base^b^	3.74 ± 2.27	2.81 ± 2.20	< 0.001
Average pain at mid^c^	3.82 ± 2.29	2.81 ± 2.17	< 0.001
Average pain at apex^d^	4.08 ± 2.32	2.83 ± 2.18	< 0.001

Data are expressed as mean ± standard deviation

PNB, periprostatic nerve block; SD, standard deviation; TRUS, transrectal ultrasound; VAS, visual analog scale

^a^Mean and SD of the average of 12 sites for each individual VAS pain scores

^b^Mean and SD of the average of 4 base sites for each individual VAS pain scores

^c^Mean and SD of the average of 4 mid sites for each individual VAS pain scores

^d^Mean and SD of the average of 4 apex sites for each individual VAS pain scores

### Mean profile plots

**Fig. 2:**
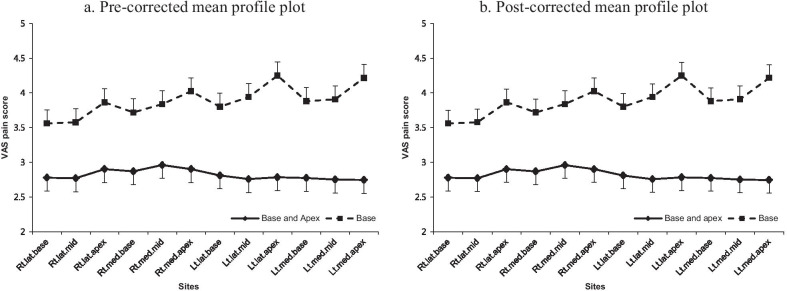
Mean profile plot of VAS pain scores for each of the 12 core biopsy sites. **a** VAS pain score before adjustment for age and VAS pain score at probe insertion. **b** VAS pain score after adjustment for age and VAS pain score at probe insertion. Lat, lateral; Lt, left; Med, medial; Rt, right; TRUS, transrectal ultrasound; VAS, visual analog scale

**Fig. 3:**
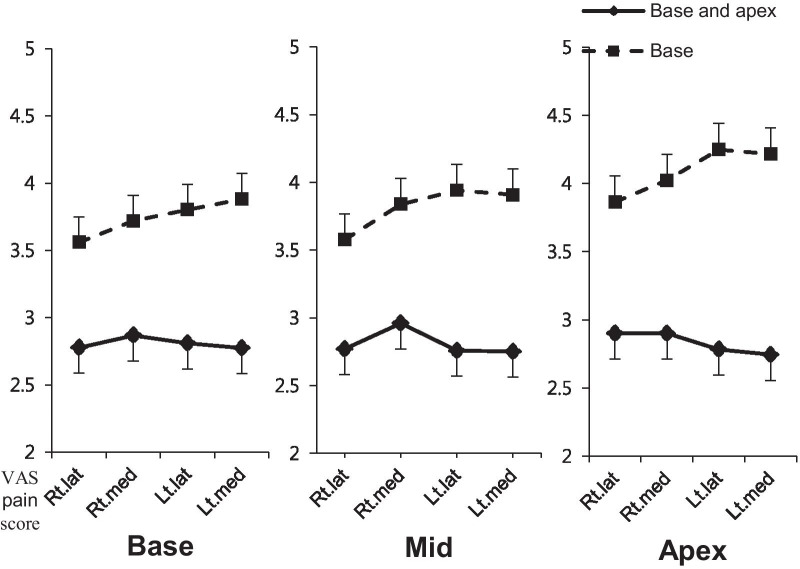
Mean profile plot of VAS pain scores in base, mid, and apex regions. VAS pain scores for each of the 12 core biopsy sites were divided into base, mid, and apex regions. Adjustments were made for age and the VAS pain score at probe insertion. Lat, lateral; Lt, left; Med, medial; Rt, right; TRUS, transrectal ultrasound; VAS, visual analog scale

Older age (*p* = 0.029) and higher VAS scores at probe insertion (*p* = 0.002) were correlated with higher individual VAS scores in each of the 12 core biopsy sites, but a history of prostate biopsy (*p* = 0.616) and DRE (*p* = 0.131) were not. The mean profile plots show the results before and after adjustment for age and VAS pain scores at probe insertion; the VAS pain scores for each of the 12 core biopsies were not significantly different pre- and post-correction for these confounding variables (Fig. 2). The VAS pain scores were significantly higher in the base-only PNB group than in the base and apex PNB group (*p <* 0.001; Fig. 2). A comparison across biopsy locations in the base-only PNB group (after categorization of the 12 sites into the base, mid, and apex regions) indicated that VAS pain scores increased from the base to apex (*p <* 0.001; Fig. 3). The biopsy of the medial sites was marginally, but significantly, more painful than that of the lateral sites within the base region in the base-only PNB group (*p =* 0.091); no significant differences were observed between the medial and lateral sites in the mid (*p =* 0.134) or apex (*p =* 0.392) regions. Pain scores at each site also gradually increased as the biopsy proceeded in the base-only PNB group (*p <* 0.001).

### Complications

There were two cases (1.3 %) of vasovagal syncope in the base-only PNB group, and both the cases recovered without medical therapy. No major complications were observed in the base and apex PNB group (Table 1).

## Discussion

The main factors related to pain during prostate biopsy were identified as age, procedure duration, prostate volume, and lithotomy position [6, 13–15]. Our results confirmed that age and procedure duration are associated with pain due to TRUS-guided prostate biopsy. The administration of a base PNB in addition to an apex PNB resulted in lower VAS pain scores when compared to the base-only PNB, and this result is consistent with that reported in previous studies [6]. We compared VAS pain scores for the base-only PNB across the 12 individual core biopsy sites and observed higher levels of pain at the medial and apex sites than at the lateral and base sites. This novel finding suggests that a potential strategy for the mitigation of severe pain during the biopsy procedure is the administration of an apex PNB, in addition to the base PNB.

The conventional method for pain control during a TRUS-guided prostate biopsy consists of the combination of a PNB and IRLG. A meta-analysis of 26 articles (comprising 36 randomized controlled trials) concluded that the combination of IRLG and PNB is effective and safe [16]. The PNB achieves different degrees of pain control depending on the lidocaine injection site. Previous studies have reported the VAS pain score collected after the procedure to range between 3.37 and 4.97 for the base-only PNB [9, 17, 18]. The VAS pain score of 3.88, as observed in our study, was the mean value of scores collected at each 12 core biopsy sites and was consistent with previously reported values.

The prostate apex is known to be a particularly painful site in TRUS-guided prostate biopsies because of the dominant somatic nerve supply to the region below the dentate line [6]. Our results indicated that the apex PNB was more painful than the prostate base PNB. The apex injection blocks the sensitive somatic nerve branch of the inferior rectal nerve (arising from the pudendal nerve), which is contained in the distal part of the dentate line; this region is penetrated by the needle during PNB or biopsy [19]. Thus, the anatomy of this region may explain the results of a prior study, which reported the increased efficacy of an additional apex lidocaine injection for the PNB method [6]. This previous study reported that the administration of both a base and apex PNB reduced the overall VAS pain score, which is consistent with our results.

Rafael et al. reported that pain in each core biopsy site became more severe as the procedure proceeded [14]. The patients experienced pain when the first puncture was performed, which made them more nervous. This, in turn, resulted in their tendency to physically avoid the pain upon expectation of an ensuing puncture, which potentially led to more severe pain during the subsequent core biopsy [6, 20]. In our study, the base-only PNB group showed similar results. Interestingly, a correlation between procedure progression and VAS pain scores was not observed in the base and apex PNB group. Thus, our results demonstrate the potential benefit of the base and apex PNB, in terms of pain control over the duration of the biopsy procedure.

The results of the present study indicated that the degree of pain relief was also dependent on the site of the lidocaine injection. In the base-only PNB group, the VAS pain score during the apex biopsy was higher than that during the base biopsy. When VAS pain scores were compared between the lateral and medial sites, a marginally significant difference was observed only during the base biopsy. Since the prostatic base is broader than the apex, it can be inferred that a greater distance between the biopsy location and lidocaine injection site is associated with increased pain for the base-only PNB. Our results are consistent with those reported by previous studies that investigated the relationship between prostate volume and the efficacy of local anesthesia [15, 16].

The most recent NCCN guidelines have recommended that prostate magnetic resonance imaging (MRI) should be performed before biopsy to reduce unnecessary prostate biopsies based on the efficacy of radiologic diagnosis [21–24]. Prostate MRI is often effective in identifying cancerous lesions at the anterior and apex sites, which are usually not supported in random prostate biopsies [25]. However, the optimal local anesthetic methods for MRI-/TRUS-guided biopsy in the anterior and apex sites are unknown due to the lack of previous studies on pain variation across different regions. Nevertheless, our results may aid the determination of appropriate local anesthetic protocols in patients with apex lesions during MRI-/TRUS-guided biopsy.

The present study has some limitations that should be acknowledged. First, a retrospective design was used, and the study was conducted at a single center; therefore, the results are sensitive to selection bias. Second, the apex PNB was more painful than the base PNB, and may have adverse events. The results may also have been affected by different total doses of lidocaine injection. We are currently in the process of conducting a prospective, well-controlled, randomized multicenter trial to confirm the results of this study.

## Conclusions

Prostate cancer is one of the most prevalent cancers, with rapidly increasing incidence worldwide. TRUS-guided random prostate biopsy is a painful procedure and requires appropriate local anesthesia. The results of this study indicated that core biopsy of the apex site was more painful than was the base site in patients who received the base-only PNB. The administration of an additional apex PNB provided better overall pain control.

## Data Availability

The datasets used and analyzed during the current study are available from the corresponding author on reasonable request.

## Abbreviations

DRE: Digital rectal exam
IRLG: Intrarectal lidocaine gel
MRI: Magnetic resonance imaging
NCCN: National Comprehensive Cancer Network
PNB: Periprostatic nerve block
PPB: Pelvic plexus block
PSA: Prostate-specific antigen
SD: Standard deviation
TRUS: Transrectal ultrasound
VAS: Visual analog scale
